# Pedigree-Based Description of Three Traditional Hungarian Horse Breeds

**DOI:** 10.3390/ani12162071

**Published:** 2022-08-14

**Authors:** Renáta Klein, János Oláh, Sándor Mihók, János Posta

**Affiliations:** 1 Department of Animal Husbandry, Institute of Animal Science, Biotechnology and Nature Conservation, Faculty of Agricultural and Food Sciences and Environmental Management, University of Debrecen, H-4032 Debrecen, Hungary; 2 Doctoral School of Animal Science, University of Debrecen, H-4032 Debrecen, Hungary; 3 Farm and Regional Research Institute of Debrecen, University of Debrecen, H-4032 Debrecen, Hungary

**Keywords:** pedigree quality, gene origin, inbreeding, genetic diversity

## Abstract

**Simple Summary:**

The most important purpose of animal conservation programs is to maintain genetic variability. The Furioso-North Star, the Gidran, and the Nonius are indigenous Hungarian horse breeds from the Mezőhegyes Stud. In the last century, the role of the horses was changed, the technical innovations and motorization replaced them, so the population size and the genetic variability of these breeds were reduced. Nowadays these breeds are endangered. The aims of this study were to give information about the current breeding population and support breeder associations during their gene conservation work. The pedigree quality, generation intervals, probability of gene origin, and inbreeding were evaluated. We found that breeds had a large bottleneck effect during breeding history. The level of inbreeding was measured with different methods, such as Ballou’s, Wright’s, and Kalinowski’s coefficient. Most of the current inbreeding coefficient was the result of previously fixed alleles for each breed. Effective population size was also estimated, and the status of the breeds was found to be not critical according to FAO criteria.

**Abstract:**

The Mezőhegyes Stud was founded in 1784 where three different horse breeds were developed: the Furioso-North Star, the Gidran, and the Nonius. These breeds were based on the same mare population, but each breed had different utilization purposes. Our aim was to analyze the pedigree information of these three indigenous breeds. The genealogical information was traced back from the actual breeding population back to the founder animals, and the final database contained more than 47,000 horses. The reference populations were defined as the registered breeding animals in 2019. The complete generation equivalent was 16.45 for the Gidran breed, 15.18 for Furioso-North Star, and 12.64 for Nonius, respectively. Due to the utilization of English Thoroughbred during the breeding history, the average maximum generations were close to 36 generations for each breed. The average relatedness was approximately 4%. The average Wright’s inbreeding coefficient was the highest for the Nonius breed (5.59%). Kalinowski’s decomposition of inbreeding showed that inbreeding is originated mainly from the past; the current fixation of alleles was higher for the Nonius horse breed. There was a reasonable bottleneck effect for each breed. The estimated effective population sizes suggest that there is no problem with the maintaining of Mezőhegyes horse breeds.

## 1. Introduction

The Furioso-North Star, the Gidran and the Nonius horse breeds were developed in Mezőhegyes Stud, which was among the most modern farms in the 19^th^ century in the Austro-Hungarian Monarchy. Pedigree recording started in Mezőhegyes Stud, in 1785. In the beginning, each herd was distinguished based on their color. The breeds’ separation began around in 1855 and the National Studbook Committee started their separate studbooks from 1885. Each breed was denominated after the first high impact founder stallion. Furioso and North Star were English Thoroughbreds, Siglavy Gidran was desert Arabian, and Nonius was an Anglo-Norman stallion. These breeds were originated basically from the same mare population, but each breed had a different type of use as well. English Thoroughbred stallions were allowed during history in each breed, and also Arabians in the Gidran breed. The restriction was in the breeding strategy, that only mares were selected from such matings, and they were mated to purebred stallions. The broodmare population was stabilized below 2–300 broodmares per breed during their breeding history. Due to the political changes after World War I and II, the population has been widespread among Carpathian countries. Due to political changes in Hungary, breeding associations were founded in 1989 for each breed. There are small breeding stocks in Croatia, Poland, Romania, Serbia as well as in Slovakia from these breeds. According to FAO criteria [1], these breeds have been endangered in Hungary since 2004 by the decision of the Hungarian Parliament and are important genetic resources in the Carpathian Basin.

The Gidran and the Furioso-North Star were riding horses and the Gidran was used as a light cavalry horse, whereas the Furioso-North Star was characterized as a heavier type. The breeding aim of the Nonius breed was originally to produce draft and driving horses especially for military purposes. Currently, Furioso-North Star and Gidran horses are competing mostly in show-jumping competitions, and the Nonius breed popular in the horse driving.

Pedigree analysis has an important role in gene conservation, as it could give information about the individual’s ancestors and collateral relatives. The results of these measurements will suggest appropriate strategies to monitor mating and manage genetic variability to enlarge the selection basis useful for a selection program [2]. Pedigree-based relationship coefficients are often used to characterize the level of inbreeding in a population when effects of the inbreeding depression begin to be detectable.

Several publications in the past few years have described the genetic variability of different breeds of horses based on pedigree analysis. There were studies about world breeds such as English Thoroughbred [3], Arabian [4,5,6], and Quarter Horse [7] breeds. Furthermore, local breeds such as Old Kladruber [8], Posavina [9], and Pura Raza Español [10,11] were analyzed as well. There are also several research studies about pony and small horse breeds as well, such as Asturcón pony [12], Hucul [13], Slovak Sport Pony [14], and Skyros pony [15]. The relationship between coat color and genetic diversity was also evaluated [16] using the technique.

The aims of the current research were to evaluate the quality of the pedigree, generation interval, gene origin, inbreeding, and effective population size of these three indigenous Hungarian horse breeds.

## 2. Materials and Methods

The pedigree information was given by the Furioso-North Star Horse Breeding Association, the Kisbéri and Gidran Horse Breeding Association, and the Nonius Horse Breeding Association. The studbook data of the registered Furioso-North Star, Gidran, and Nonius populations up to 2019 were analyzed. The genealogical information was traced back from present horses back to the founder animals.

The following information was stored for each animal in the database: name of the individual, name of the sire, name of the dam, birth date, sex, and breed. There were the pedigree data of 47,682 animals in the developed database, including 3544 Gidran (916 stallions and 2628 mares), 11,753 Nonius (4739 stallions and 7014 mares), and 4708 Furioso-North Star (2146 stallions and 2561 mares) horses. The active populations in 2019 were chosen as reference populations for each breed.

The pedigree analysis was carried out using Endog v4.8 software (Madrid, Spain) [17]. Before the analysis, the database was checked by the Pedigree Viewer v6.5 (Armidale, Australia) software [18]. The distinct measurements for inbreeding were estimated using Grain v2.2 (Wien, Austria) [19] software.

The following parameters were used to describe the populations:

- Quality of the pedigree [20]:
  ◦ equivalent complete generations (GenEqu)—computed as the sum over all known ancestors of the terms computed as the sum of (1/2)^n^ where n is the number of generations separating the individual to each known ancestor
  ◦ number of complete generations (GenCom)—the furthest generation where all ancestors of the individual are known
  ◦ the maximum number of generations (GenMax)—the number of generations separating the individual from its furthest ancestor
- Generation Interval (GI)—the average age of the parents at the birth of their offspring kept for reproduction [21]. Generation intervals were estimated on four different pathways separately in this study: sire-to-daughter, sire-to-son, dam-to-daughter, and dam-to-son ways. The four pathways were compared pairwise for each using independent samples *t*-test, for each breed
- Probability of gene origin
  ◦ Number of founders (Nf)—Number of animals with unknown parents [3]
  ◦ Number of ancestors (Na)—the minimum number of individuals in the pedigree, which explains the total genetic variability in the reference population [3]
  ◦ Effective number of founders (fe)—the number of equally contributing founders that would be expected to produce the same genetic diversity as in the population under study [22]
  ◦ Effective number of ancestors (fa)—the marginal contributions of ancestors that would be expected to produce the same genetic diversity as in the population under study [22]
- Inbreeding level, average relatedness
  ◦ Wright method (F_Wright)—The probability that the two alleles at any locus in an individual are identical by descent. The formula used for the calculation of the inbreeding coefficient is:
    (1) $$F_{X}=\sum {\left(\frac{1}{2}\right)}^{n+n^{\prime }+1}\times \left(1+F_{A}\right)$$
    where *A* is the common ancestor in the chains of origin of the father and mother of the individual *X*, *n* and *n′* are the number of generations between the individual *X* and the common ancestor *A* on the paternal side (*n*) and the maternal side (*n′*), and *F_A_* is the inbreeding coefficient of the common ancestor [23]
  ◦ Ballou method (F_Ballou)—The probability that any allele in an individual has been homozygous in previous generations at least once [24]
  ◦ Kalinowski method (F_Kal) and Kalinowski new method (F_Kal_new)—The F_Kal represents that part of the genome where alleles are currently in identical by descent status and have also been identical by descent in an ancestor of the animal at least once. The inbreeding coefficient was split into two parts following the method of Kalinowski et al. [25], whether identical alleles were inbred in the past (F_Kal) or became inbred in recent generations (F_Kal_new)
  ◦ Ancestral History Coefficient (AHC)—quantifies the frequency that a randomly taken allele has undergone identical by descent status in the past [19]
  ◦ Average relatedness (AR)—the probability that a randomly drawn allele from the population, belongs to a given individual [26]
- Effective population size (Ne)—the number of breeding horses that would lead to the same increase in inbreeding, as observed in the population under study, if they would contribute equally to the next generation [27]
  ◦ individual increase in inbreeding (Ne_f) [26]
  ◦ regression on equivalent generations (Ne_reg) [26]
  ◦ log regression on equivalent generations (Ne_log) [26]

## 3. Results

### 3.1. Quality of the Pedigree

The more complete pedigree allows more precise results because there are fewer unknown ancestors. The estimates of inbreeding level highly depend on the pedigree depth and completeness [28]. Table 1 presents the main indexes for pedigree quality.

The complete generations equivalent varied between 12.64 and 16.45. The Gidran was the highest and the Nonius was the lowest.

The mean number of complete generations was the highest (6.1) for the Gidran population; there was an individual with nine known full generations in this reference group. At least six generations of pedigree information were completely known for the 39.9% of the animals in the three reference populations.

Due to the English Thoroughbred background, the pedigrees were really deep, starting in the early 1700s years. The average maximum generations were close to 36 generations for each breed. There was a Nonius mare with a 43 generations-long pedigree. In the active populations, almost every horse (99.3%) had at least a 36 generations-long pedigree.

### 3.2. Generation Interval

The estimated generation intervals are shown in Table 2. The four pathways were compared pairwise for each breed, using independent samples *t*-test. The sire pathways were significantly longer (*p* < 0.05) compared to dam-related pathways for each breed. The sire-to-son and sire-to-daughter pathways did not differ from each other. The dam-to-son and dam-to-daughter pathways were also similar, except for the Gidran breed where the dam-to-son pathway was significantly (*p* < 0.05) lower than dam-to-daughter pathway.

### 3.3. Probability of Gene Origin Based Parameters

Table 3 shows parameters describing the genetic variability of the reference populations. The fe values were ranged between 95 and 99, whereas fa values were nearly the same for the Gidran and the Nonius population. The ratio of the effective number of ancestors and effective the number of founders shows that all populations were affected by a bottleneck effect. The ratio was higher for the Furioso-North Star breed whereas it was quite similar for the Gidran and the Nonius breeds. These lower values of the Gidran and Nonius breed might be the result of the smaller breeding population and the fewer used English Thoroughbred stallions during the breeding history.

Table 4 presents the 10 most influential ancestors’ contribution to the genetic variability. These 10 ancestors accounted for nearly 55% of the genetic diversity for the Gidran and the Nonius breeds and approximately 40% for the Furioso-North Star breed. The most important ancestor covered 13.8% of the genetic diversity within the Nonius population and 10.3% within the Gidran population. In the Furioso-North Star population, the most important ancestor accounts for 7.751% of the genetic diversity. These numbers also strengthen the idea that the Furioso-North Star breed is more diverse compared to Gidran and Nonius breeds.

Table 5 gives information from the genetic variability of the three reference populations. The active Gidran population could be described with only 138 individuals. These values were 311 and 239 in the other two breeds. Only 9–9 animals covered 50% of the genetic variability for Gidran and Nonius breeds, respectively. In the Furioso-North Star population, that was a bit higher at 17 horses. The tendencies of these numbers are in agreement with the numbers presented in Table 4.

### 3.4. Inbreeding Level, Average Relatedness

The three reference populations are quite small, so mating of related horses is not avoidable. Computation of ancestral inbreeding coefficients could provide information that alleles identical by descent for the first time or were already homozygous. Table 6 summarizes the inbreeding information about the three breeds. There were close to 70% inbred horses within each population. The percentage of inbred animals was the highest for Gidran horses in the reference population and in the total population as well.

The inbreeding was calculated in different ways. Ancestral inbreeding coefficients were also estimated to determine if inbreeding was happening presently or in the past. The Wright inbreeding coefficient was the highest for the active Nonius population. The inbreeding coefficient usually increases over time especially in small and closed populations where the mating of related individuals is unavoidable. The lowest inbreeding was estimated for the Furioso-North Star breed. The Ballou’s ancestral inbreeding coefficient and the A_HC_ were higher than other estimated parameters especially in the Gidran breed. The lowest values were calculated for the Nonius breed in both ways. The mean estimates for F_Kal and F_Kal_new were much lower than Ballou’s coefficient. The estimated F_Kal_new values were smaller than F_Kal, so inbreeding originated mostly from the past. The Kalinowski’s inbreeding coefficients were the lowest for the Furioso-North Star population.

### 3.5. Effective Population Size

The effective population size is a crucial parameter for planning strategies to define and protect endangered animals. As none of the studbooks were closed, the average inbreeding might differ year-by-year (and not continuously increase), and other estimations of the effective population size might be also interesting. Table 7 gives information about the Ne, which was estimated in three different ways. Due to the unequal contribution of the breeding individuals to the next generation, the effective population size is always smaller compared to the exact population size. The effective population size based on inbreeding (Ne_f) was much higher than the other parameters. It was the lowest (101.96) for the Nonius population compared to the other two breeds.

The regression-based effective population sizes (Ne_reg, Ne_log) were quite close to each other for each breed. Contradictory to inbreeding-based effective population size, the Gidran and the Nonius were close to each other while the lowest coefficients were estimated for the Furioso-North Star.

## 4. Discussion

The average number of the complete generations was higher for the Gidran breed than it was estimated for the Slovakian Shagya-Arabian and Lipizzan populations [14] whereas lower values were calculated for the Furioso-North Star and Nonius breeds. Our estimated values were higher than what was reported for the Sardinian Anglo-Arab horses [29]. Our deep pedigrees allow reliable estimations for further parameters. The average equivalent complete generations for the Furioso-North Star and Gidran breeds were close to other breeds with long breeding history such as Lipizzan [30] and English Thoroughbred [3]. The Nonius population’s value was lower than it was reported in previous studies but close to the Noriker breed’s [31]. The numbers of complete generations were in alignment with what was reported for a composite horse breed, whereas the equivalent complete generations were higher [16]. Due to the English Thoroughbred background of these breeds, several pedigrees started in the early 1700s. The long pedigree information allows us to estimate reliable parameters to describe the three breeds.

As it might be expected, the sire pathways were longer than dam pathways. This could be the result of the selection method of the breeding stallions. In general, all breeding individuals started reproduction in young age. Breeding stallions receives their breeding license in older age, while the broodmares usually started foaling at four years old. The sire pathways were quite similar than it was reported for Arabian horses [4,14]. The dam pathways were in agreement with estimations for Mallorquí [32], the Brazilian Sport Horse [33], and the Maremmano [34,35] breeds. Our findings exceeded the calculated values reported for the Posavac breed [9]. There is a usual demand during gene conservation that breeding animals should be used as long as it is possible. This breeding strategy results in longer generation intervals along with less genetic progress but could help to prevent gene loss. This could also help to prevent decreased genetic diversity, which might be an important demand during gene conversation.

The calculated fe values for the three breeds were quite similar and were close to estimations for the Dutch Harness Horse population [36]. Our computed numbers were higher than findings for Andalusian [2], Arabian [4,37], and Lipizzan [38] horses. The background of these differences might be the different breeding techniques. The traditional breeding of Mezőhegyes horse breeds allowed the usage of English Thoroughbred (and Arabian in Gidran) stallions while the cited breeds have closed pedigree. Breeds having opened studbooks had higher fe values compared to our findings [39,40,41]. This suggests that small populations have a higher risk of gene loss and are more endangered compared to worldwide known breeds. The effective numbers of ancestors were quite similar for the Gidran and the Nonius population. These estimations were in alignment with Arabian [4] and Shagya-Arabian [14]. The more flexible breeding regulations of the Furioso-North Star breed had resulted a higher fa value, which is close to the findings for the Holstein breed [42]. Our numbers were higher than the fa values reported for English Thoroughbred [3] and Lipizzan [38] horses, while higher values than our findings were reported for Sport Horse breeds [34,41]. These findings also strengthen the differences among opened, bottom-closed, and closed studbooks.

The fa/fe ratios showed a bottleneck effect for each breed. The reason for the huge genetic loss could be found in the history of the breeds. There was high loss of breeding animals after World War I and II as only a few broodmares and breeding stallions remained at the original stud. Due to technical innovations and motorization, military as well as agricultural usage also decreased. The genetic loss of the Furioso-North Star was almost equal to Lusitano [43] and Maremanno [35] horses, whereas the estimations for Gidran and Nonius were quite close to Old-Kladruber [8] and Quarter [7,40].

Concentration of genetic variability was different across the breeds under study. The bottom-closed studbook of the Gidran and Nonius breeds might have resulted in the first ten ancestors covering more than 50% of total genetic variability. The genetic concentration was lower for the Furioso-North Star breed as the first ten ancestors covered less than 40% of the total variability. The tendency for fa50, fa60, and fa70 were similar for the Gidran and Nonius breeds, though from fa80 the Gidran breed had lower genetic diversity. Furioso-North Star had the highest values for all different categories; the final number is almost three times higher compared to the Gidran breed.

The level of the inbreeding coefficient in the population is crucial for maintaining the genetic variability of the breed. The ratio of inbred horses within the reference populations were similar for each breed. The AR values were higher than half of the Wright coefficient, so there was mating of related individuals in each breed. The Wright’s coefficient was around 5% for each breed. These estimations were somewhat lower than the mean inbreeding of the composite Hispano-Arabian horse [16] and Franches-Montagnes population [44], which were in agreement with the Mallorquí [32] and the Arabian [4] breeds. The probability that an allele has been homozygous in previous generations was near 30% for the Nonius breed and above 30% for Furioso-North Star and Gidran breeds. These values were higher than it was estimated for the Hungarian Hucul population [45] and for the Holsein breed [42].

Kalinowski’s and Kalinowski’s new formula were not frequently researched in horse breeding. According to the definition, F_Kal deals with alleles that were homozygous because they have met in the past, and only includes the ancestral inbreeding of relationship. So, F_Kal for an individual remains zero when its F_Wrigth is zero. The ancestral proportion of the inbreeding was quite similar for the breeds under study. This might be the result of the common maternal origin and the usage of English Thoroughbred stallions in the past. Kalinowski’s decomposition of inbreeding showed that the current fixation of alleles was higher for the Nonius among the analyzed horse breeds. The estimated value for the Nonius showed that inbreeding was quite high in the recent generation and was in agreement with Old Kladruber horses [46]. Furioso-North Star and Gidran horses had lower present inbreeding, and those values were aligned with estimations for Holstein horses [42].

A population with low effective population size has a higher probability of extinction [4]. The critical Ne value is 50; if the estimated value is lower, it suggests a problem with the maintainability of the population [47]. The coefficient was measured in three ways and all of our results were above that level. They were quite similar in the case of Ne_reg and Ne_log for each breed, whereas the inbreeding-based effective population size (Ne_f) was higher in each case. The inbreeding-based effective population sizes of Furioso-North Star and Gidran horses were higher than those estimated for various colors of the Hispano-Arabian horses, while only the estimated value for Nonius was smaller than the value reported for grey Hispano-Arabian horses [16]. Our numbers were higher than what was reported in previous studies for Holstein [42], Hungarian Hucul [45], and Maremanno [35] horses. The genetic diversity was the lowest for the Nonius breed based on the Ne_f, and Furioso-North Star was the least endangered breed.

## 5. Conclusions

The average value of equivalent complete generations allows the estimation of reliable pedigree-based population genetic parameters for the reference populations. Each breed had a large bottleneck effect, but effective population size showed that inbreeding depression could be avoided in the analyzed populations. Most of the current inbreeding coefficient was the result of previously fixed alleles for each breed.

## Figures and Tables

**Table 1:** Pedigree completeness is the reference populations.

	Furioso-North Star	Gidran	Nonius
Actual breeding stock	646	367	521
GenEqu	15.18	16.45	12.64
GenCom	4.69	6.10	4.95
GenMax	36.56	36.32	36.53

**Table 2:** Estimated generation intervals for the populations (years).

Pathways	Furioso-North Star	Gidran	Nonius
Sire-to-son	12.64 (n = 6058) ^a^	12.77 (n = 3984) ^a^	12.54 (n = 5322) ^a^
Sire-to-daughter	12.63 (n = 13,263) ^a^	12.77 (n = 9163) ^a^	12.41 (n = 12,267) ^a^
Dam-to-son	10.75 (n = 5269) ^b^	10.83 (n = 3601) ^c^	10.72 (n = 4586) ^b^
Dam-to-daughter	10.88 (n = 12,087) ^b^	11.10 (n = 8587) ^b^	10.63 (n = 10,882) ^b^
Average	11.78 (n = 36,677)	11.93 (n = 25,335)	11.61 (n = 33,057)

Different superscript letters show significant difference (*p* < 0.05).

**Table 3:** Founders and ancestors in the reference populations.

	Furioso-North Star	Gidran	Nonius
Nf	2874	1725	2019
Na	311	138	239
fe	99	99	95
fa	43	24	22
fa/fe	0.43	0.24	0.23

**Table 4:** Contribution of the ancestors to the gene pool of the reference populations (%).

	Furioso-North Star	Gidran	Nonius
1st ancestor	7.751	10.337	13.812
2nd ancestor	6.123	8.019	8.048
3rd ancestor	4.980	7.868	6.545
first 10 ancestors	39.815	54.729	55.702

**Table 5:** Concentration of genetic variability in the reference populations.

	Furioso-North Star	Gidran	Nonius
fa50	17	9	9
fa60	27	13	12
fa70	41	18	19
fa80	62	26	30
fa90	107	42	59
fa100	311	138	239

**Table 6:** Inbreeding in the reference populations.

	Furioso-North Star	Gidran	Nonius
Inbred animals (%)	76.00	78.20	68.27
AR	4.08	3.88	3.68
F_Wright	4.31	4.95	5.59
F_Ballou	34.39	39.30	28.87
F_Kal	3.07	3.48	3.29
F_Kal_new	1.24	1.48	2.40
A_HC_	66.90	73.64	45.45

**Table 7:** Effective population size in the reference populations.

	Furioso-North Star	Gidran	Nonius
Ne_f	164.65	152.50	101.96
Ne_reg	70.35	77.67	77.67
Ne_log	69.17	77.47	78.25

## Data Availability

The data presented in this study are available on request from the corresponding author.
